# Ticks and Tick-Borne Pathogens Associated with Dromedary Camels (*Camelus dromedarius*) in Northern Kenya

**DOI:** 10.3390/microorganisms9071414

**Published:** 2021-06-30

**Authors:** Dennis Getange, Joel L. Bargul, Esther Kanduma, Marisol Collins, Boku Bodha, Diba Denge, Tatenda Chiuya, Naftaly Githaka, Mario Younan, Eric M. Fèvre, Lesley Bell-Sakyi, Jandouwe Villinger

**Affiliations:** 1 International Centre of Insect Physiology and Ecology (*icipe*), Nairobi P.O. Box 30772-00100, Kenya; gdennoh89@gmail.com (D.G.); tatendachiuya@gmail.com (T.C.); 2 Department of Biochemistry, Jomo Kenyatta University of Agriculture and Technology, Nairobi P.O. Box 62000-00200, Kenya; 3 Department of Biochemistry, School of Medicine, University of Nairobi, Nairobi P.O. Box 30197-00100, Kenya; ekanduma@uonbi.ac.ke; 4 Institute of Infection, Veterinary and Ecological Sciences, University of Liverpool, Liverpool L3 5RF, UK; Marisol.Collins@liverpool.ac.uk (M.C.); Eric.Fevre@liverpool.ac.uk (E.M.F.); L.Bell-Sakyi@liverpool.ac.uk (L.B.-S.); 5 Directorate of Veterinary Services, County Government of Marsabit, Marsabit P.O. Box 384-60500, Kenya; bokubodha@gmail.com (B.B.); dengediba@gmail.com (D.D.); 6 International Livestock Research Institute, Nairobi P.O. Box 30709-00100, Kenya; N.githaka@cgiar.org; 7 Food and Agriculture Organization of the United Nations (FAO), Programme & Operational Support to Syria Crisis, UN cross-border hub, Gaziantep 27010, Turkey; mario.younan@fao.org

**Keywords:** dromedary camels, ticks, heartwater, zoonosis, tick-borne pathogens, *Anaplasma*, *Coxiella*, *Ehrlichia*, *Rickettsia*

## Abstract

Ticks and tick-borne pathogens (TBPs) are major constraints to camel health and production, yet epidemiological data on their diversity and impact on dromedary camels remain limited. We surveyed the diversity of ticks and TBPs associated with camels and co-grazing sheep at 12 sites in Marsabit County, northern Kenya. We screened blood and ticks (858 pools) from 296 camels and 77 sheep for bacterial and protozoan TBPs by high-resolution melting analysis and sequencing of PCR products. *Hyalomma* (75.7%), *Amblyomma* (17.6%) and *Rhipicephalus* (6.7%) spp. ticks were morphologically identified and confirmed by molecular analyses. We detected TBP DNA in 80.1% of blood samples from 296 healthy camels. “*Candidatus* Anaplasma camelii”, “*Candidatus* Ehrlichia regneryi” and *Coxiella burnetii* were detected in both camels and associated ticks, and *Ehrlichia chaffeensis*, *Rickettsia africae*, *Rickettsia aeschlimannii* and *Coxiella* endosymbionts were detected in camel ticks. We also detected *Ehrlichia ruminantium*, which is responsible for heartwater disease in ruminants, in *Amblyomma* ticks infesting camels and sheep and in sheep blood, indicating its endemicity in Marsabit. Our findings also suggest that camels and/or the ticks infesting them are disease reservoirs of zoonotic Q fever (*C. burnetii*), ehrlichiosis (*E. chaffeensis*) and rickettsiosis (*R. africae*), which pose public health threats to pastoralist communities.

## 1. Introduction

Kenya is home to over 3 million camels, representing about 6% of Africa’s camel population [1,2]. In the northern parts of Kenya and the Horn of Africa, camel production is a major source of livelihood [1,3]. Since the 1960s, camel populations in this region have continued to increase despite numerous challenges brought about by climate change [2]. In response to increasingly frequent droughts, pastoralist communities that did not previously keep camels have started rearing them to supplement or replace income from cattle production [1]. In comparison with other ruminant livestock, camels are biologically and physiologically adapted to survive better in arid and semi-arid environments [4,5]. They provide a reliable source of meat and milk, even during dry seasons when production from other livestock species such as goats, sheep and cattle becomes insufficient [1]. Camels also play a role as beasts of burden [5].

Despite the economic importance and resilience of camels under harsh climatic conditions, camel production is constrained by pests, vector-borne diseases and parasites. Common haematophagous ectoparasites of camels, specifically in Marsabit County, northern Kenya, include biting flies (e.g., *Tabanus, Stomoxys* and *Haematopota*), the camel fly or camel ked *Hippobosca camelina* [6,7] and ticks, which are important disease vectors. While biting flies as mechanical vectors for trypanosomes in camels have been the subject of research for decades [7], very little is known about tick-borne pathogens circulating among camels in northern Kenya.

Tick-borne pathogens (TBPs) cause emerging and re-emerging diseases in Africa and beyond [8,9]. They are transmitted to animals and humans through tick bites and constitute major constraints to livestock production in Kenya [10]. Ticks are vectors of a wide range of pathogens, including viruses, bacteria and protozoa that can infect domestic and wild animals and humans [11,12,13]. These pathogens may cause bacterial diseases such as Q fever, rickettsiosis, ehrlichiosis and anaplasmosis, protozoal diseases such as babesiosis and theileriosis and viral diseases such as Crimean–Congo hemorrhagic fever [11]. Ticks of the genus *Hyalomma* are most commonly associated with camels and are known vectors of *Theileria*, *Babesia*, *Anaplasma*, *Rickettsia* and *Ehrlichia* spp. [14,15,16]. Other genera of ticks infesting camels in Kenya include *Rhipicephalus* and *Amblyomma* [3,17].

In Kenya, most of the studies undertaken on ticks and TBPs of livestock have been limited to cattle, sheep and goats; camels remain understudied. Climate change, as well as the extensive movement of camels and other ruminant livestock across semi-arid counties and the northern borders of Kenya with neighbouring countries such as Somalia, Ethiopia and South Sudan, potentially contribute to shifts in the distribution of ticks and TBPs in the region. An *Ehrlichia* sp. with a DNA sequence close to *E. ruminantium* was found in ticks infesting camels in herds affected by an outbreak of heartwater-like disease in dromedary camels in the Moyale Constituency of Marsabit County; the disease occurred in most of North Kenya’s camel keeping region and caused significant losses in adult animals in 2016 [18]. The present study, in which blood samples and ticks were collected from dromedary camels and co-herded sheep in Marsabit County, northern Kenya, was carried out as part of a wider investigation into the possible involvement of *E. ruminantium* and heartwater in this novel camel disease. Co-herded sheep were included in the wider study as indicators for the presence of *E. ruminantium* infection in an area because they develop high and long-lasting levels of serum antibodies following exposure [19,20]; the results from the serological investigation in camels and sheep will be presented in a separate publication. Here we report the results from the morphological identification of tick species infesting healthy camels and co-herded sheep, molecular detection and the characterization of various TBPs in the ticks and host blood. To the best of our knowledge, this is the first detailed molecular study on tick species infesting camels in Kenya and on TBPs in blood and ticks from these camels and their co-grazing sheep.

## 2. Materials and Methods

### 2.1. Study Area

The study was conducted in February 2020 in Marsabit County in northern Kenya, with an area of ~66,923 km^2^ about 543 km north of Nairobi [21]. Marsabit County is located between longitudes 37°57’ and 39°21’ East and latitudes 02°45’ and 04°27’ North and borders Wajir and Isiolo Counties to the East, Turkana County to the West, Samburu County to the South and Ethiopia to the North. Marsabit County experiences extreme temperatures with minimum and maximum temperatures ranging from 16 °C to 39 °C [22]. The long wet season is from March to May, while the short wet season is from October to December [21]. Most of the County is located 300–900 m above sea level with an average annual rainfall ranging from below 150 mm to just over 1000 mm. Marsabit County is home to pastoralist camel keepers who rely on mobile livestock production for their livelihoods.

**Figure 1:**
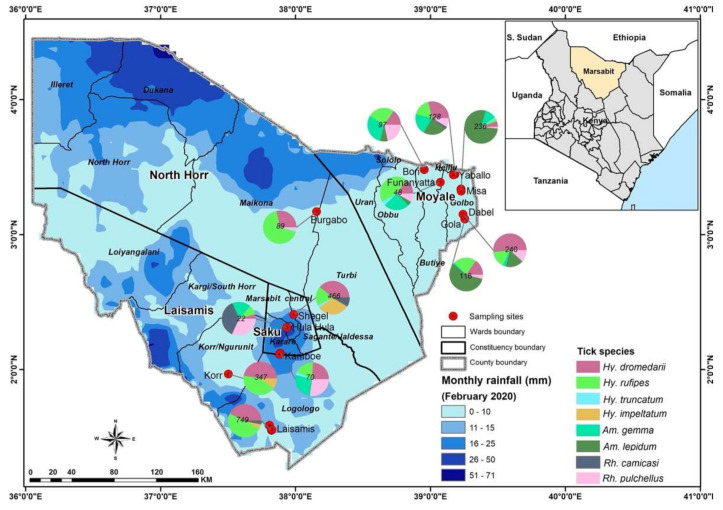
Sampling sites in Marsabit County, Kenya, showing the spatial distribution of tick species collected from camels and co-grazing sheep. Maps were created using the open-source software, QGIS v.3.10.6. Pie charts indicate numbers of ticks collected at sampling sites.

Blood samples and ticks were collected from healthy dromedary camels and from co-grazing sheep at 12 sites: Laisamis, Korr, Hula Hula, Kamboe, Shegel, Burgabo, Gola, Misa, Funanyatta, Dabel, Yabalo and Bori (Figure 1). The wells located at these sites are important watering points for camels and other livestock.

### 2.2. Ethical Approval

This study was undertaken in strict adherence to the experimental guidelines and procedures approved by the University of Nairobi Biosafety, Animal Use and Ethics Committee (REF: FVM BAUEC/2019/200) and Kenya’s National Commission for Science, Technology and Innovation (REF: NACOSTI/P/19/72855/27325). Animals were handled carefully to cause minimum discomfort. The camel pastoralists were informed about the study and, thereafter, sampling of camels was conducted after receiving verbal consent as most herders were unable to read or write in addition to the language barriers that required translation by our field assistants selected from the community.

### 2.3. Collection of Blood Samples and Ticks from Camels and Co-Herded Sheep

Sample collection from 296 healthy camels and from 77 healthy co-herded sheep was conducted during the dry season from February 2020 to March 2020. Co-grazing sheep were sampled as sentinel animals for a parallel serological study of *E. ruminantium* antibody levels in this combined livestock cohort, as part of the overarching study investigating the role of heartwater and other TBPs in camel diseases in Kenya. Four millilitres of blood were collected from individual animals via jugular venepuncture using vacutainer needles (18 gauge) and EDTA vacutainer tubes. Blood samples were kept under cold chain (4–10 °C) for up to 6 h before being preserved in liquid nitrogen for transportation to the Martin Lüscher Emerging Infectious Diseases (ML-EID) laboratory at *icipe*, Nairobi, for the molecular detection of TBPs.

Serrated forceps held firmly over the tick scuta and mouthparts were used to gently remove all visible ticks attached to the skin of sampled camels and sheep. Ticks were placed in cryovials and kept under cold chain (4–10 °C) for up to 2 h prior to preservation in liquid nitrogen for transportation to the ML-EID molecular biology laboratories for further analysis.

### 2.4. Morphological Identification of Ticks

Ticks were identified to species level using taxonomic keys [23]. The morphological features used for tick identification included the colour and ornamentation of the scutum, shape, size and distribution of punctations and grooves and colour of legs. The ticks were staged under a Stemi 2000-C microscope (Zeiss, Oberkochen, Germany) and photographed using a digital microscope connected to an Axio-cam ERc 5s camera (Zeiss). Fully-engorged ticks were removed during tick identification and excluded from subsequent analysis to minimise vertebrate host DNA in nucleic acid extractions. Ticks were pooled into groups of one to eight individuals based on species, host, sampling site and date of collection.

### 2.5. Extraction of DNA from Whole Ticks, Tick Leg Tissues and Blood

Two representative adult ticks from each of the eight tick species identified from camels were selected for molecular confirmation of identity. Legs were plucked from individual ticks for genomic DNA extraction. For TBP screening, whole ticks were first frozen in liquid nitrogen before homogenising them in 1.5-mL microfuge tubes containing 150 mg of 0.1-mm and 750 mg of 2.0-mm yttria-stabilised zirconium (YSZ) oxide beads (Glen Mills, Clifton, NJ, USA) and 100 µL of 1 × PBS using a Mini-Beadbeater-16 (BioSpec, Bartlesville, OK, USA) for 1 min. The ISOLATE II Genomic DNA extraction kit (Bioline, London, UK) was used to extract DNA from the leg tissues selected for tick identification and from homogenised whole tick and blood samples for pathogen screening following the manufacturer’s instructions.

### 2.6. Molecular Identification of Ticks

In order to confirm findings of the morphological identification of tick species, we used the extracted tick genomic DNA in PCRs targeting fragments of the cytochrome oxidase subunit I (COI), 12S ribosomal (r)RNA and 16S rRNA genes (Table 1). The PCRs were performed in 10-μL reaction volumes including 2 μL 5× HOT FIREPol^®^ Blend Master Mix (Solis BioDyne, Tartu, Estonia), 0.5 μL of 10 µM forward and reverse primers (Table 1) and 25 ng of DNA template in a ProFlex PCR systems thermocycler (Applied Biosystems, Foster City, CA, USA). The following conditions were used for amplification: Initial denaturation at 95 °C for 15 min followed by 35 cycles of denaturation at 95 °C for 30 s, annealing (at 55 °C for 16S rRNA and COI, at 48 °C for 12S rRNA) for 30 s, extension at 72 °C for 1 min and a final extension at 72 °C for 10 min. Successful PCR amplification of target amplicons was determined by resolving 5 μL of the PCR products by electrophoresis in 1.5% (*w/v*) agarose gels containing ethidium bromide and DNA fragments visualised under ultraviolet light using a Kodak Gel Logic 200 Imaging System (SPW Industrial, Laguna Hills, CA, USA). The remaining volumes of positive PCR amplicons were purified using ExoSAP-IT (Affymetrix, Santa Clara, CA, USA) according to the manufacturer’s protocol and sequenced by Macrogen Inc. (Amsterdam, The Netherlands).

### 2.7. Molecular Detection of TBPs

In order to screen the DNA extracts of blood and ticks from camels and sheep for TBPs of the genera *Anaplasma*, *Babesia*, *Coxiella*, *Ehrlichia*, *Rickettsia* and *Theileria*, we conducted high-resolution melting (HRM) analysis of PCR products obtained using genus-specific primers (Table 1). The analysis was carried out in Rotor-Gene Q (QIAGEN, Hannover, Germany), Mic qPCR (Bio Molecular Systems, Upper Coomera, Queensland, Australia) and Quant Studio 3 Real-Time PCR System (Applied Biosystems, Foster City, CA, USA) thermocyclers. The primer pairs *Ehrlichia*16S and *Anaplasma*JV were used to amplify 200-bp and 300-bp fragments of *Ehrlichia* and *Anaplasma* 16S rRNA genes, respectively. Samples with unique *Ehrlichia* and *Anaplasma* 16S rRNA amplicon HRM profiles were re-amplified using longer primers targeting 16S rRNA (PER1, PER2) for *Ehrlichia* [31] and EHR16SD-1492R for *Anaplasma* [32,33]. *Theileria* and *Babesia* were amplified using primers targeting the 18S ribosomal gene (RLB_F and RLB_R) [35]. Rickettsial 16S rRNA genes were amplified using primers Rick-F and Rick-R [27]. *Rickettsia*-positive samples were re-tested using rickettsial outer membrane protein B (*ompB*) gene primers (28).

The PCRs were performed in 10-µL volumes including 2 μL HOT FIREPol^®^ EvaGreen^®^ HRM mix (Solis BioDyne, Tartu, Estonia), 0.5 μL of 10 µM forward and reverse primers and 25 ng of template DNA. For no-template controls, 1 μL nuclease-free water was used as a template. DNA samples of “*Ca*. Anaplasma camelii”, “*Ca*. Ehrlichia regneryi”, *Theileria parva* and *Rickettsia africae* from earlier studies [6,18,36,37] were used as positive controls. The PCR cycling conditions included an initial enzyme activation at 95 °C for 15 min; 10 cycles of denaturation at 94 °C for 20 s, step-down annealing from 63.5 °C to 53.5 °C (decreasing by 1 °C per cycle) for 25 s and extension at 72 °C for 30 s; 25 cycles of denaturation at 94 °C for 25 s, annealing at 50 for 20 s and extension at 72 °C for 30 s; and a final extension at 72 °C for 7 min. A 3 min hold at 72 °C was included after PCR cycling before HRM analysis by gradually increasing the temperature from 75 to 90 °C with fluorescence acquisitions after 2 s at 0.1 °C increments [34]. All samples with unique melt profiles were purified with an ExoSAP-IT PCR Product Cleanup kit (Affymetrix, Santa Clara, CA, USA) and submitted for Sanger sequencing by Macrogen (Amsterdam, The Netherlands). Chromatogram files were imported into the Geneious Prime software v. 2020.2.2 (created by Biomatters, Auckland, New Zealand) in which they were trimmed, edited and aligned to generate consensus sequences.

### 2.8. Phylogenetic Analysis

Nucleotide sequences obtained in this study were queried against known sequences in the GenBank nr database (http://www.ncbi.nlm.nih.gov/, accessed on 15 May 2021) using BLAST to confirm their identity and relation to existing deposited sequences [38]. Study sequences were then aligned with related tick or pathogen sequences available in the GenBank nr database using the MAFFT plugin in Geneious Prime software version 2020.2.2 [39]. Maximum-likelihood phylogenies were constructed using PhyML v. 3.0 with automatic model selection based on the Akaike information criterion. Tree topologies were estimated over 1000 bootstrap replicates with the nearest neighbour interchange improvements [40]. Phylogenetic trees were visualised using FigTree v. 1.4.4 [41].

### 2.9. Estimation of Tick Infection Rates

Estimated minimum infection rates (MIRs) of each of the TBPs obtained for each tick species were calculated as the number of positive pools per total number of ticks of that species tested × 100, with a conservative assumption that only one tick is positive per pathogen-positive pool.

## 3. Results

### 3.1. Morphological and Molecular Identification of Ticks

**Figure 2:**
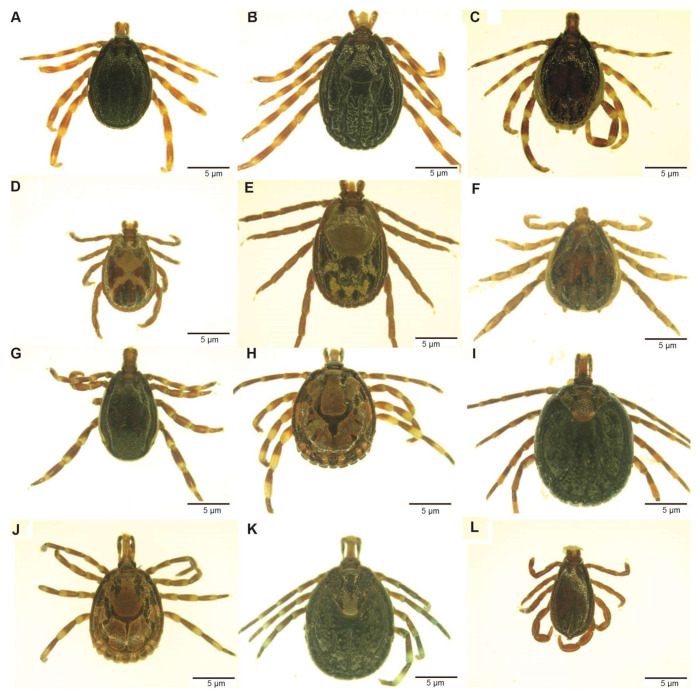
Images of adults of tick species collected from camels in northern Kenya. (**A**) *Hyalomma rufipes* male; (**B**) *Hy. rufipes*, female; (**C**) *Hyalomma impeltatum*, male; (**D**) *Rhipicephalus pulchellus*, male; (**E**) *Rh. pulchellus*, female; (**F**) *Hyalomma dromedarii*, male; (**G**) *Hyalomma truncatum*, male; (**H**) *Amblyomma lepidum*, male; (**I**) *Am. lepidum*, female; (**J**) *Amblyomma gemma*, male; (**K**) *Am. gemma*, female; (**L**) *Rhipicephalus camicasi*, male. The images were staged under a Stemi 2000-C microscope (Zeiss, Oberkochen, Germany) after thawing from liquid nitrogen and photographed using a digital microscope connected to an Axio-cam ERc 5s camera (Zeiss).

A total of 2610 adult ticks removed from camels were morphologically identified as *Hyalomma dromedarii*, *Hyalomma rufipes*, *Hyalomma impeltatum*, *Hyalomma truncatum*, *Amblyomma lepidum*, *Amblyomma gemma*, *Rhipicephalus pulchellus* and *Rhipicephalus camicasi*. *Hyalomma* was the most prevalent genus and comprised three quarters (75.7%) of all adult ticks collected from camels, followed by *Amblyomma* (17.6%) and *Rhipicephalus* (6.7%). Tick infestation was low in sheep, from which we collected 86 adult ticks and identified them as *Rh. camicasi*, *Am. gemma*, *Am. lepidum*, *Rh. pulchellus* and *Hy. rufipes*. *Rhipicephalus* (53.5%) was the dominant genus sampled from sheep, followed by *Amblyomma* (45.4%) and *Hyalomma* (1.2%). Figure 1 shows the total numbers of ticks and the proportions of each species collected at each sampling site. Table 2 summarises the number of tick species collected from camels and co-herded sheep in northern Kenya. Photomicrographs of representative specimens of the eight tick species identified are shown in Figure 2.

**Figure 3:**
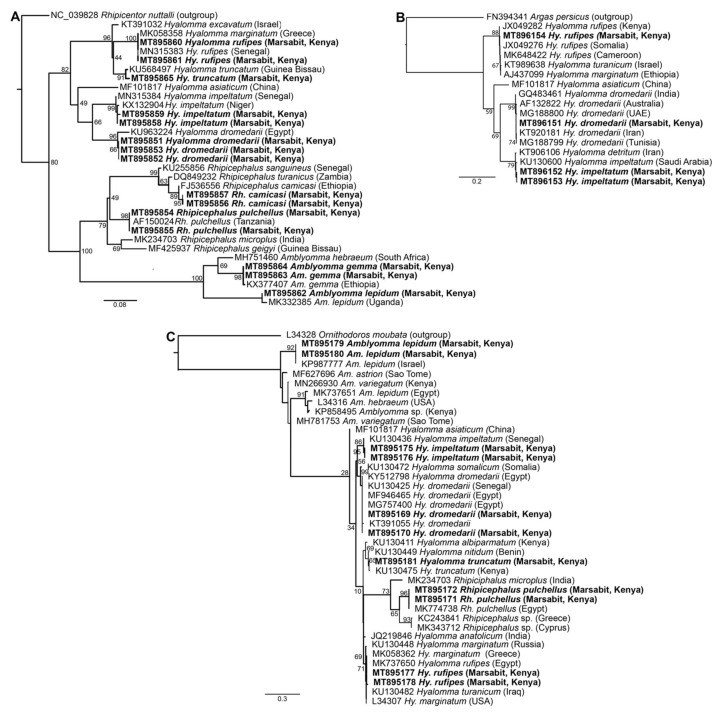
Maximum likelihood phylogenetic trees of representative gene sequences from samples of ticks collected from camels in Northern Kenya. (**A**) 12S rRNA, (**B**) COI mitochondrial and (**C**) 16S rRNA gene sequences. Sequences obtained from this study, with their GenBank accession numbers, are noted in bold. Bootstrap values at the major nodes are of percentage agreement among 1000 bootstrap replicates. The branch length scale represents substitutions per site. Trees are rooted to outgroup sequences (indicated in brackets; top sequence of each tree).

BLASTn analysis of *Am. gemma, Am. lepidum, Rh. camicasi, Rh. pulchellus, Hy. dromedarii, Hy. impeltatum, Hy. truncatum* and *Hy. rufipes* sequences obtained in this study showed identities ranging from 99 to 100% with reference sequences from the GenBank nr database (Table S1). Molecular identification based on partial 12S rRNA, 16S rRNA and COI gene sequences obtained from 15 representative samples was consistent with morphological identification and confirmed the wide diversity of tick species collected from camels (Figure 3). The 12S rRNA and 16S rRNA molecular identification was more informative due to more consistent amplification as we were able to amplify COI sequences from only four of the tick samples. All tick sequences obtained in this study have been deposited in GenBank (COI gene accessions MT896151-MT896154; 12S rRNA gene accessions MT895851-MT895865; 16S rRNA gene accessions MT895169-MT895181).

### 3.2. Tick-Borne Pathogens Detected in Camel and Sheep Blood

**Figure 4:**
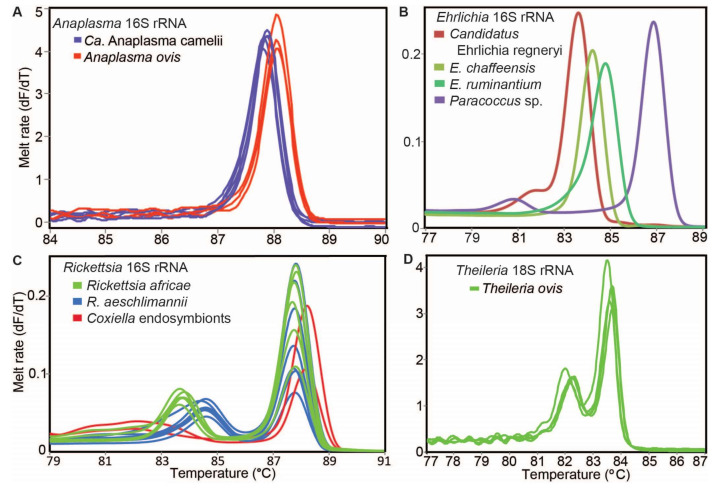
Representative melt rate profiles of tick-borne pathogens in tick samples collected from camels and sheep in northern Kenya. PCR-amplicon melt rates are represented as change in fluorescence with increasing temperature (dF/dT) of (**A**) *Anaplasma* 16S rRNA, (**B**) *Ehrlichia* 16S rRNA, (**C**) *Rickettsia* 16S rRNA and (**D**) *Theileria* 18S rRNA gene amplicons.

**Figure 5:**
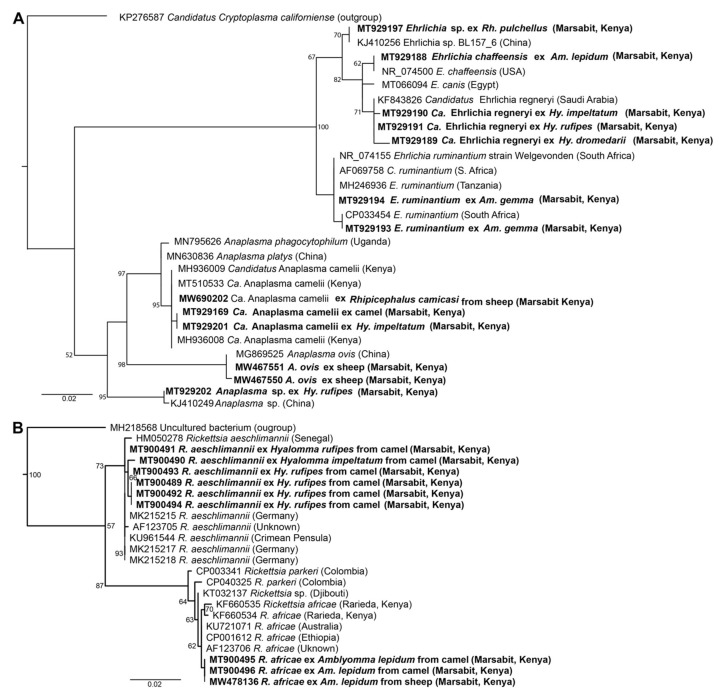
Maximum likelihood phylogenetic trees of (**A**) 1030-bp *Anaplasma* spp. and 451-bp *Ehrlichia* spp. 16S rRNA sequences and (**B**) 857-bp *Rickettsia* spp. ompB sequences. Sequences amplified from blood and ticks infesting camels in northern Kenya in this study are indicated in bold. Bootstrap values at the major nodes are of percentage agreement among 1000 bootstrap replicates. The branch length scale represents substitutions per site. Trees are rooted to outgroup sequences (indicated in brackets; top sequence of each tree).

We detected tick-borne pathogens with distinct HRM profiles (Figure 4) that shared ≥99% identity with sequences from other recognised TBP species in GenBank (Table 3). Three bacterial species, “*Candidatus* Anaplasma camelii”, “*Candidatus* Ehrlichia regneryi” and *Coxiella burnetii*, were detected in camels using genus-specific primers, with infection rates of 78.7%, 14.5% and 3.4%, respectively (Table 3). “*Candidatus* A. camelii” 16S rRNA (1030 bp), “*Ca.* E. regneryi” 16S rRNA (451 bp) and *C. burnetii* transposon-like IS1111 (687 bp) gene sequences were successfully amplified from camel blood and characterised by sequencing. The *C. burnetii* sequences (GenBank accessions MT900497-MT900501,) shared 100% identity with *C. burnetii* reference sequences with GenBank accessions DQ379976, KT954146 and MT268529. *Rickettsia, Theileria* and *Babesia* pathogens were not detected in the blood collected from camels. In blood collected from co-herded sheep, we detected *E. ruminantium* (100% nucleotide sequence identity to *E. ruminantium* strain Welgevonden GenBank accession NR_074155), *Ehrlichia chaffeensis* (100% identity to *E. chaffeensis* strain Arkansas, GenBank accession NR_074500), *Theileria ovis* (100% identity to *T. ovis* GenBank accession MN712508) and *Anaplasma ovis* (100% identity to *A. ovis*, GenBank accession MG869525) (Table 4, Figure 5A,B). *Anaplasma ovis* and *T. ovis* were not detected in camels.

### 3.3. Tick-Borne Pathogens and Endosymbionts Detected in Ticks

We screened 858 tick pools from camels for *Ehrlichia*, *Anaplasma*, *Rickettsia*, *Coxiella*, *Babesia* and *Theileria* species. In ticks sampled from camels, we detected 451-bp 16S rRNA gene sequences of *E. ruminantium*, “*Ca.* E. regneryi”, *E. chaffeensis* and an *Ehrlichia* sp. Sequence similarities to reference sequences and to TBPs identified in camel herds of the study region in 2016 are indicated in Table 3 and the maximum likelihood phylogenetic relationships are shown in Figure 5A. We detected an *E. ruminantium* sequence*,* identical to that found in sheep blood, in *Am. gemma* and *Am. lepidum*; “*Ca.* E. regneryi” was detected in all three species of *Hyalomma*; *E. chaffeensis* (100% identity to *E. chaffeensis* strain Arkansas, GenBank accession NR_074500) was detected in *Am. lepidum* ticks; and an *Ehrlichia* sp. (100% identity to *Ehrlichia* sp. MN726921, detected in a *Hyalomma anatolicum* tick in Pakistan) was detected in *Hy. rufipes*, *Am. gemma*, *Rh. camicasi* and *Rh. pulchellus* ticks from different camels in different herds. Additionally, using the primer pair Ehrlichia16S F and Ehrlichia 16S R, we amplified short (163 bp) sequences identified as *Paracoccus* sp. (99% identity to *Paracoccus* sp. BRM2, GenBank accession KP003988, isolated from Tunisian phosphogypsum) in *Amblyomma, Hyalomma* and *Rhipicephalus* spp. collected from camels at five different sites (Table S3).

We detected identical *C. burnetii* sequences in camel blood and *Hy. dromedarii*, *Hy. rufipes* and *Rh. pulchellus* ticks. Additionally, we detected *Coxiella* endosymbionts in *Am. gemma*, *Am. lepidum* and *Rh. pulchellus* ticks using the Rick16S primers.

We detected “*Ca.* Anaplasma camelii” in all the species of *Hyalomma*, *Amblyomma* and *Rhipicephalus* spp. identified in this study; an *Anaplasma* sp. sequence in *Hy. rufipes*; *Rickettsia aeschlimannii* in *Hyalomma* and *Rhipicephalus* spp.; and *Rickettsia africae* in the two *Amblyomma* spp. identified in this study as shown in Table 3 and Figure 5B. In ticks sampled from sheep, we detected *E. ruminantium* (100% identity to *E. ruminantium* strain Welgevonden NR_074155) in *Amblyomma* spp.; *R. africae* in *Amblyomma* spp.; *Theileria ovis* in *Rhipicephalus* spp.; *Anaplasma ovis* in *Amblyomma* and *Rhipicephalus* spp.; and “*Ca*. A. camelii” in *Rh. camicasi* (Table 4). The distributions of ticks and pathogens according to the sampling sites are shown in Table S2. We detected three pathogens, “*Ca.* Anaplasma camelii” (21.3%), “*Ca.* Ehrlichia regneryi” (3.4%) and *C. burnetii* (0.3%), in both blood and ticks from the same individual camels. Among sheep, we detected *E. ruminantium*, *A. ovis* and *T. ovis* in both ticks and blood from the same individual sheep (Table S3).

All sequences generated in this study have been submitted to GenBank under the following accessions: MT900489-MT900496 and MW478135-MW478138 for *R. aeschlimannii* and *R. africae,* MT900497-MT900501 for *C. burnetii*, MT929189-MT929192 for “*Ca.* E. regneryi”, MT929193-MT929195 and MW467546 for *E. ruminantium*, MT929188 for *E. chaffeensis*, MT929196-MT929197 for *Ehrlichia* sp., MT929199-MT929201, MT929169-MT929177 and MW690202 for “*Ca.* A. camelii”, MT929202 for *Anaplasma* sp., MW541904-MW541911 for *Coxiella* endosymbionts, MW467555-MW467561 for *T. ovis* and MW467547-MW467552 for *A. ovis*. The maximum likelihood phylogenies of all pathogen sequences obtained in this study along with sequences from previously characterized and closely-related TBPs from GenBank are represented in Figure 5.

## 4. Discussion

This study provides critical insight on the diversity and abundance of tick species on camels and co-herded sheep in northern Kenya and the TBPs in ticks and blood from these animals. Tick species on camels identified in this study confirm earlier reports on camel ticks in North Kenya [17]. We also report for the first time that *Hy. impeltatum* ticks parasitise camels in Kenya. Notably, we found a diversity of ticks and tick-borne microorganisms associated with camel herds distinct from those recently identified on cattle in western Kenya [42]. We identified four TBPs, *R. africae* (in sheep ticks), *E. ruminantium* (in camel ticks, sheep ticks and sheep blood), *E. chaffeensis* (in camel ticks and sheep blood) and *C. burnetii* (in camel ticks and camel blood), that are of major economic, animal health and/or human health importance [11,13,43]. Information on tick species diversity, ecology and distribution will help improve the understanding of disease dynamics [44] and is a prerequisite for any future prophylaxis or control measures.

### 4.1. Species Diversity of Ticks Associated with Camels and Co-Herded Sheep in Northern Kenya

We identified eight epidemiologically-important tick species from three different genera, *Hyalomma*, *Amblyomma* and *Rhipicephalus,* parasitising camels and co-herded sheep. These tick genera have been reported to infest a broad range of vertebrate host species and transmit several important pathogens, including viruses, bacteria and protozoa of medical and veterinary importance [12,45].

The most prevalent tick species sampled from camels were *Hy. dromedarii* (35.21%) and *Hy. rufipes* (31.03%). *Hyalomma dromedarii* is considered to be the main tick species parasitising dromedary camels [16,17,46] and is the principal vector of *Theileria* spp. of camels in Egypt [47]. *Hyalomma dromedarii* may play a role in the epidemiology or transmission of emerging and re-emerging diseases such as rickettsioses [48,49,50], viruses such as Crimean–Congo haemorrhagic fever virus (CCHFV) and *C. burnetii* (responsible for zoonotic Q fever) [51,52]. A high prevalence of *Hy. dromedarii* has been reported in camels found in arid and hyper-arid regions of Kenya [17], Saudi Arabia, Sudan, Egypt, Iran and Tunisia, with infestation rates ranging between 49% and 89% [15,16,45,53,54,55]. This tick can also infest other livestock, such as cattle, goats, sheep and horses [56,57], though we did not find this species on sheep co-herded with camels in the present study. *Hyalomma rufipes*, found on both camels and sheep, is known to be a vector of CCHFV as well as of *R. aeschlimannii, Anaplasma marginale, Rickettsia conorii* and *Babesia occultans* [58,59,60]. We also found, for the first time in Kenyan camels, *Hy. impeltatum*, which has previously been found on dromedary camels in Iran and northern Sudan [46,55,57]. *Hyalomma impeltatum* is known to have a wide range of animal hosts including buffalo, cattle and sheep [16,61] and has the potential to transmit CCHFV [62].

We found *Am. lepidum* and *Am. gemma* ticks on both camels and sheep. The economic importance of *Amblyomma* spp. ticks has long been recognised due to their ability to transmit multiple diseases to humans and animals [11]. They are the major vectors of *E. ruminantium*, which is the causative agent of heartwater disease in sub-Saharan Africa (SSA) and some Caribbean and Indian Ocean islands [11,63,64,65]. Other tick species found on camels in our study include *Hy. truncatum*, *Rh. camicasi* and *Rh. pulchellus*; the latter two species were also found on sheep. Our report of *Rh. camicasi* infesting sheep and camels in northern Kenya extends knowledge about the geographic range and dynamics of this tick species in Kenya. Since most of these tick species have the potential to transmit diseases such as heartwater, anaplasmosis and Q fever, domestic animals and humans in the region may be exposed to a variety of tick-borne diseases.

### 4.2. Tick-Borne Bacteria Identified in Ticks, Camels and Co-Herded Sheep in Northern Kenya

Our findings show that *E. ruminantium*, *E. chaffeensis*, “*Ca*. E. regneryi”, *C. burnetii*, “*Ca*. A. camelii”, *A. ovis* and *T. ovis* are circulating among ticks from camels and sheep in the study area. The findings also show occurrence of distinct TBPs, with some overlap, in blood and ticks from camels and sheep in the study area.

*Ehrlichia ruminantium* was detected in *Am. gemma* and *Am. lepidum* ticks sampled from both camels and co-herded sheep in this study and in sheep blood, but not in camel blood. The bacterium is known to infect macrophages, neutrophils and vascular endothelial cells of ruminant hosts and is a major cause of livestock loss in SSA [63]. The absence of the pathogen in blood samples is not surprising considering the fact that *E. ruminantium* is mainly found in endothelial cells and can only be rarely detected in the bloodstream, except during clinical heartwater [66,67]. Our finding of *E. ruminantium*, which causes heartwater disease in ruminants, in *Amblyomma* ticks feeding on camels supports the recent reports on their potential impact on SSA camel populations [18,68], although it remains unknown if camels are susceptible to infection with *E. ruminantium*. Our findings in combination with the identification of *Ehrlichia* sp. with a DNA sequence close to *E. ruminantium* in Moyale Constituency, which is part of the study area in 2016 [18], and the isolation of the pathogen from *Amblyomma* spp. in eight districts across Kenya [69] suggest that there is continuous circulation of *E. ruminantium* across the country. Since 2016 and during the entire study period in 2020, no clinical heartwater cases were reported from camels, sheep and goats in Marsabit (Boku Bodha, unpublished observations). This is an indication that *E. ruminantium* may be endemic in Marsabit County. However, there is lack of information on the role of camels in the epidemiology of ehrlichiosis.

*Ehrlichia chaffeensis* DNA was detected in *Am. lepidum* ticks from camels and in blood from sheep. *Ehrlichia chaffeensis*, an emerging TBP, is known to cause human monocytic ehrlichiosis in humans [70]. Recent studies have found *E. chaffeensis* in *Haemaphysalis leachi* ticks collected from dogs in Uganda [71], *Rhipicephalus sanguineus* from dogs in Cameroon [72], *Amblyomma hebraeum* collected from both cattle and sheep in South Africa [73] and questing *Amblyomma eburneum* ticks in Kenya [34], which suggests that diverse tick species may vector this pathogen. To our knowledge, this is the first detection of *E. chaffeensis* in *Am. lepidum* ticks collected from dromedary camels. Our finding of *E. chaffeensis* in *Am. lepidum* ticks collected from camels and in blood from co-grazing sheep in northern Kenya suggests that this pathogen is actively circulating in the study area. Further investigation on the epidemiology of this pathogen is required.

We detected “*Ca.* E, regneryi” in camel blood and in *Hy. rufipes*, *Hy. dromedarii* and *Hy. impeltatum* ticks removed from camels, but not in other tick species feeding on camels. “*Candidatus* E. regneryi” is a novel *Ehrlichia* sp. first described in Saudi Arabia [74]. During an outbreak of heartwater-like disease in Moyale Constituency of Marsabit County in 2016, “*Ca.* E. regneryi” was found in blood from one camel that had reportedly recovered from the disease; however, the pathogen was not detected in ticks and blood from a severe clinical case of heartwater-like disease in a recumbent camel [18]. Our findings suggest that *Hyalomma* spp. are important vectors of the bacterium. “*Candidatus* E. regneryi” is phylogenetically closely related to *Ehrlichia canis* [74]. It is interesting to note that we did not detect the pathogen in blood and ticks from co-herded sheep, which suggests that it may be specific to camels. The pathogen was detected in apparently healthy camels, which suggests that the parasite in circulation is non-pathogenic and this is in line with the observations made in 2016 that the pathogen was not found in diseased camels [18]. Our findings suggest that camels are asymptomatic carriers of “*Ca*. E. regneryi” and further investigation into its pathogenicity, key vectors and zoonotic potential is required.

For Q fever, which is caused by *C. burnetii*, the association between camel exposure, seroprevalence in camels and human Q fever infections is well documented from Chad [52], and a recent study in Somalia found that *C. burnetii* infections were common in camel ticks [75]. Q fever is one of the most widespread neglected zoonosis worldwide with the highest seroprevalence rates recorded in female camels with a history of abortion [76]. *Coxiella burnetii* infection has been found in *Hy. dromedarii* and *Hy. impeltatum* ticks from camels in Tunisia [77]. While ticks facilitate a sylvan life cycle of *C. burnetii* in reservoir animals, domestic animals and humans are most commonly infected by contaminated aerosols [78]. We found *C. burnetii* in camel blood and in *Hy*. *rufipes*, *Hy. dromedarii* and *Rh. pulchellus* ticks from camels, which indicates that dromedary camels could be an additional reservoir species for this pathogen. In Laikipia, Kenya, just south of this study’s geographic focus, 18.6% of camels have been found to have been exposed to *C. burnetii* by seropositivity [79]. The acute *C. burnetii* prevalence documented for healthy camels in this study (3.4%) is comparable to the prevalence in clinically asymptomatic cattle (4.3%) with a history of previous abortions and reproductive problems [80]. *Coxiella burnetii* is known to cause infections in a wide range of species, including domestic animals, birds and reptiles [29]. Ticks have been shown to transmit *C. burnetii* experimentally and could play a role as reservoirs in maintaining the bacterium in the environment between outbreaks due to their very long lives [81]. A study in Algeria suggested that *Hyalomma* spp. ticks could facilitate the transmission of *C. burnetii* among dromedary herds [51]. The detection of *C. burnetii* in *Rhipicephalus* and *Hyalomma* spp. corroborates previous reports on the same findings in Kenya [82,83,84] and Senegal [85]. Our results demonstrate that camels and their associated ticks in northern Kenya constitute an important epidemiological reservoir of *C. burnetii*, which increases human exposure and zoonotic risk of Q fever infection for camel-keeping communities, veterinarians and abattoir workers in the area. Antibodies against *C. burnetii* have been found in significant numbers of livestock handlers, indicating exposure to the pathogen [86,87]. Given the potential impact of *C. burnetii* on camel reproduction and the zoonotic risk for public health, further studies are required to better understand the role of camels in the epidemiology of Q fever.

*Coxiella* endosymbionts were detected in *Am. lepidum*, *Am. gemma* and *Rh. pulchellus* ticks using *Rickettsia* 16S rRNA gene primers. To the best of our knowledge, this is the first study to register *Coxiella* endosymbionts in ticks collected from northern Kenya. Previous studies in Kenya have revealed the presence of *Coxiella* endosymbionts in ticks collected from Busia [37], the Maasai Mara National Reserve [36] and the coastal region [34]. *Coxiella* endosymbionts assist in blood meal processing and egg production by providing the tick host with essential micronutrients and macronutrients [88,89]. The roles of these *Coxiella* endosymbionts in ticks are still not clear and require further investigation. *Coxiella burnetii* and *Coxiella* endosymbionts have different transmission routes and infectiousness, even though they are closely related [81]. Understanding the role of *Coxiella* endosymbionts in ticks may advance our understanding of Q fever.

We detected “*Ca*. A. camelii” in 78.7% of blood samples from 233 apparently healthy camels, indicating presence of an asymptomatic healthy carrier state. This high prevalence of “*Ca*. A. camelii” was found in camels carrying *Amblyomma* ticks with *E. ruminantium* infection rates between 5.2% and 12.4%. The fact that no heartwater cases were reported, observed or suspected in these camels throughout the study contradicts the notion that immunosuppression by “*Ca*. A. camelii” may be a contributing factor in development of clinical heartwater-like disease in camels, a hypothesis which could not be ruled out entirely during the 2016 outbreak [18]. The present study corroborates previous findings of “*Ca*. A. camelii” in blood from healthy camels in Kenya (6,18) and in other dromedary camel populations [53,90,91,92]. Carrier status or persistence in the host is an important strategy for successful pathogen transmission to ticks and for developing resistance against the reinfection of hosts [93]. The high prevalence of “*Ca*. A. camelii” in healthy camels is an indication of endemic stability and/or that the bacterium is non-pathogenic. We detected “*Ca.* A. camelii” in all eight tick species removed from these 233 camels, with infection rates in tick pools ranging from 2.7% to 8.5%. We also detected “*Ca*. A. camelii” in one *Rh. camicasi* tick collected from co-grazing sheep, but not in sheep blood. Interestingly, “*Ca.* A. camelii” has also been found in hippoboscid flies (*Hippobosca camelina*) collected from camels in northern Kenya [6]. These flies can transmit “*Ca*. A. camelii” to small laboratory animals [94], indicating that hippoboscids might also play a role in the transmission of this organism. High infection rates of 88.3% found for *A. ovis* in clinically healthy sheep blood during this study suggest that sheep in northern Kenya may serve as reservoirs for this pathogen. Although *A. ovis* infection is generally a subclinical infection in small ruminants, more severe infections resulting in significant economic losses have been reported in Spain [95].

We found high infection rates for *R. africae* in *Am. gemma* (10.9%) and *Am. lepidum* (9.4%) tick pools from camels and in *Am. gemma* (14.3%) and *Am. lepidum* (16.7%) tick pools from co-herded sheep. The detection of *R. africae* in *Amblyomma* ticks collected from camels and sheep points towards the importance of camel-associated and sheep-associated *Amblyomma* ticks as significant reservoirs of zoonotic *R. africae* in North Kenya. For *R. aeschlimannii*, the infection rates in camel tick pools were 4.8% for *Rh. pulchellus*, 4.0% for *Hy. truncatum*, 2.7% for *Hy. impeltatum*, 1.1% for *Hy. rufipes* and 0.33% for *Hy. dromedarii*, respectively. These low infection rates suggest that camel-associated *Hyalomma* and *Rhipicephalus* spp. ticks represent minor reservoirs for *R. aeschlimannii.* Our findings correlate well with other studies that have predominantly detected *R. africae* in *Amblyomma* spp. and *R. aeschlimannii* in *Hyalomma* and *Rhipicephalus* spp. [59,60,96]. While *R. africae* and *R. aeschlimannii* were detected in both camels and their associated ticks in Algeria [97], we did not detect spotted fever group rickettsiae (SFGR) DNA in camel or sheep blood in the current study. The absence of SFGR may be due to the minute numbers of rickettsial organisms in the blood samples tested, the limited number of samples tested in this study or because the ticks are not actually transmitting the bacteria under normal circumstances. Camels in northern Kenya are kept close to homesteads and herds are in close association with other animals such as goats and sheep; thus, the presence of *R. africae* and *R. aeschlimannii* in ticks may present a health risk to humans. Healthcare providers in these areas should consider SFGR diseases in the differential diagnoses of patients presenting with fever of unknown origin and clinical signs compatible with rickettsioses.

We did not detect *Theileria* or *Babesia* spp. DNA in camel blood or in camel ticks. However, we did detect *T. ovis* in blood samples (80.5%) and *Rh. camicasi* ticks from healthy sheep. Similar high prevalence of *T. ovis* DNA in sheep blood has previously been reported in Ethiopia (91.9%) [98] and Sudan (88.6%) [99]; lower prevalences of 27–50% in sheep in Ghana were based on the morphological identification of piroplasms in blood smears [100]. 

We also detected *Paracoccus* sp. in *Ambylomma*, *Hyalomma* and *Rhipicephalus* spp. collected from camels; this raises the possibility of these bacteria being transmitted or harboured by ticks or by another invertebrate organism parasitising ticks. These bacteria were first associated with ticks feeding on horses at a single site in Brazil [101] and subsequently in Kenya with *Amblyomma* spp. ticks collected from livestock and tortoises at a single sample site [59], as well as with questing *Haemaphysalis concinna* ticks at two sites in Hungary [102] and *Rhipicephalus microplus* ticks removed from a collared peccary in Peru [103]. Further investigation is required to elucidate the relationship between *Paracoccus* bacteria and ticks and whether they pose any risk to animal or human health.

## 5. Conclusions

This is the first study to show tick and TBP point prevalence and infection rates among Kenyan camel herds. We found that *Hy. dromedarii* and *Hy. rufipes* are the most prevalent tick species on camels in northern Kenya and that camels are exposed to a range of TBPs. We also report for the first time that *Hy. impeltatum* ticks parasitise camels in Kenya. We report the presence of *“Ca.* E. regneryi”, *“Ca*. Anaplasma camelii” and *C. burnetii* in camel blood and *E. ruminantium, “Ca.* E. regneryi”, *E. chaffeensis, “Ca*. Anaplasma camelii”, *R. aeschlimannii, R. africae*, *C. burnetii* and *Coxiella* endosymbionts in camel ticks in northern Kenya. Some of these pathogens, such as *R. africae*, *E. chaffeensis* and *C. burnetii*, are zoonotic and therefore have a potential to cause serious illness in humans. Presence of *Coxiella* endosymbionts in ticks raises exciting questions on the role they might play in tick physiology, population dynamics and the transmission of disease-causing pathogens. We found distinct TBPs, with some overlap, in blood and ticks from camels and sheep in the study area. These findings form a basis for strategic frameworks for research and development of novel control strategies, which are necessary to protect camels from threats that TBPs may pose. Further studies are required to identify the vectors of *“Ca.* E. regneryi” and *“Ca*. Anaplasma camelii” and for determining their effects on camel health and productivity. The epidemiology of *E. ruminantium* in camels needs to be investigated further to assess the potential involvement of this pathogen in heartwater-like disease of camels.

## Figures and Tables

**Table 1:** Primers used for molecular identification of ticks and tick-borne pathogens.

Primer Name	Target Gene	Sequence (5’–3’)	Amplicon Size (bp)	Reference
**Tick COI F** **Tick COI R**	Tick COI	ATTCAACCAATCATAAAGATATTGG TAAACTTCTGGATGTCCAAAAAATCA	658	[24]
**SR-J-14199F** **SR-N-14594R**	Tick 12S rRNA	TACTATGTTACGACTTAT AAACTAGGATTAGATACCC	430	[25]
**Tick 16S** **Tick 16S**	Tick 16S rRNA	AATTGCTGTAGTATTTTGAC TCTGAACTCAGATCAAGTAG	450	[26]
**Rick-F** **Rick-R**	*Rickettsia* 16S rRNA	GAACGCTATCGGTATGCTTAACACA CATCACTCACTCGGTATTGCTGGA	364	[27]
**120–2788** **120–3599**	*Rickettsia ompB*	AAACAATAATCAAGGTACTGT TACTTCCGGTTACAGCAAAGT	856	[28]
**Trans1** **Trans2**	*Coxiella* IS1111	TGGTATTCTTGCCGATGAC GATCGTAACTGCTTAATAAACCG	687	[29]
**Ehrlichia16S F** **Ehrlichia16S R**	*Ehrlichia* 16S rRNA	CGTAAAGGGCACGTAGGTGGACTA CACCTCAGTGTCAGTATCGAACCA	200	[30]
**PER1** **PER2**	*Ehrlichia* 16S rRNA	TTTATCGCTATTAGATGAGCCTATG CTCTACACTAGGAATTCCGCTAT	451	[31]
**EHR16SD** **1492R**	*Anaplasma/Ehrlichia* 16S rRNA	GGTACCYACAGAAGAAGTCC GGTTACCTTGTTACGACTT	1030	[32,33]
**AnaplasmaJV F** **AnaplasmaJV R**	*Anaplasma* 16S rRNA	CGGTGGAGCATGTGGTTTAATTC CGRCGTTGCAACCTATTGTAGTC	300	[34]
**RLB F** **RLB R**	*Theileria*/*Babesia* 18S rRNA	GAGGTAGTGACAAGAAATAACAATA TCTTCGATCCCCTAACTTTC	460–520 bp	[35]

**Table 2:** Tick species collected from camels and co-herded sheep in Marsabit, Kenya, in February2020 to March 2020.

	From 296 Camels					From 77 Co-Herded Sheep				
Species	Male	Female	No. of Pools	No. of Ticks	Percent (%)	Male	Female	No. of Pools	No. of Ticks	Percent (%)
***Amblyomma gemma***	80	49	87	129	**4.95**	11	4	12	15	**17.44**
***Amblyomma lepidum***	186	144	120	330	**12.64**	20	4	12	24	**27.91**
***Hyalomma dromedarii***	624	295	233	919	**35.21**					
***Hyalomma*** ***rufipes***	557	253	251	810	**31.03**	1		1	1	**1.16**
***Hyalomma truncatum***	19	6	12	25	**0.96**					
***Hyalomma impeltatum***	153	68	44	221	**8.47**					
***Rhipicephalus pulchellus***	73	31	66	104	**3.98**	1		1	1	**1.16**
***Rhipicephalus camicasi***	30	42	24	72	**2.76**	22	23	22	45	**52.33**
**Total**	1734	876	858	**2610**		55	31	**48**	86	

**Table 3:** Minimum infection rates for tick-borne pathogens (TBPs) identified in ticks and blood samples collected from camels in Marsabit, Kenya (February 2020 to March 2020).

Bacterial Species (Target Gene)	TBP Detection in Ticks—Number of Positive Pools (Minimum Infection Rate)								Camels with TBPs (Infection Rate)	GenBank Accessions		Nucleotide Sequence Identity
	*Hy.* *dromedarii*	*Hy.* *rufipes*	*Hy.* *impeltatum*	*Hy.* *truncatum*	*Am.* *gemma*	*Am.* *lepidum*	*Rh.* *camicasi*	*Rh.* *pulchellus*		Study Sequences	Reference GenBank Accessions	
**No. of** **individuals**	919 ticks	810 ticks	221 ticks	25 ticks	129 ticks	330 ticks	72 ticks	104 ticks	296 camels			
**Number of tick pools**	254	251	44	12	87	120	24	66				
***Ehrlichia*** ***ruminantium*** **(16S rRNA)**					16 (12.40%)	17 (5.15%)				MT929193-MT929195	NR_074155, KU721071, CP001612	100%
***Ca.* Ehrlichia** **regneryi** **(16S rRNA)**	22 (2.39%)	46 (5.68%)	6 (2.72%)						43 (14.53%)	MT929189-MT929192	KF843826	100%
***Ehrlichia chaffeensis*** **(16S rRNA)**						2 (0.61%)				MT929188	NR_074500, NR_074501, CP007473-CP007480	100%
***Ehrlichia* sp.** **(16S rRNA)**		1 (0.12%)			1 (0.78%)		3 (4.17%)	18 (17.31%)		MT929196-MT929197	MN726921, KJ410256	100%
***Candidatus* Anaplasma camelii** **(16S rRNA)**	25 (2.72%)	27 (3.33%)	6 (2.72%)	1 (4%)	11 (8.53%)	20 (6.06%)	6 (8.33%)	7 (6.73%)	233 (78.72%)	MT929199-MT929201, MT929169-MT929177	MT510533, MK388297	100%
***Anaplasma* sp.** **(16S rRNA)**		1 (0.12%)								MT929202	KJ410248, KJ410249	100%
***Rickettsia*** ***africae*** **(*ompB*)**					14 (10.85%)	31 (9.39%)				MT900495-MT900496	KU721071, KT032136, CP0011612	100%
***Rickettsia*** ***aeschlimannii*** **(*ompB*)**	3 (0.33%)	87 (1.07%)	6 (2.72%)	1 (4.00%)				5 (4.81%)		MT900489-MT900494	MK215215-MK215218	100%
***Coxiella burnetii*** **(IS1111)**	11 (1.20%)	12 (1.50%)						5 (4.81%)	10 (3.38%)	MT900497-MT900501	DQ379976, KT954146, MT268529	100%
***Coxiella*** **endo-** **symbiont** **(16S rRNA)**					12 (9.30%)	16 (22.22%)		6 (5.77%)		MW541904-MW541911	EU143670, JX846589, MK026405	98–100%
***Paracoccus* sp.** **(16S rRNA) ^1^**	2 (0.22%)	8 (1.00%)	2 (0.90%)	1 (4.00%)	1 (0.78%)			3 (2.88%)		^2^	KP003988	99%

^1^ amplified using the primer pair Ehrlichia16S F and Ehrlichia16S R (29) (Table 1).

**Table 4:** Minimum infection rates for tick-borne pathogens (TBPs) identified in ticks and blood samples collected from sheep in Marsabit, Kenya (February 2020 to March 2020).

Bacterial Species (Target Gene)	TBP Detection in Ticks—Number of Positive Pools (Minimum Infection Rate)					Sheep with TBPs (Infection Rate)	GenBank Accessions		Nucleotide Sequence Identity
	*Hy.* *rufipes*	*Am.* *gemma*	*Am.* *lepidum*	*Rh.* *camicasi*	*Rh.* *pulchellus*		Study Sequences	Reference GenBank Accessions	
**No. of** **individuals**	1 tick	14 ticks	24 ticks	45 ticks	1 tick	77 sheep			
**No. of tick pools**	1	12	12	22	1				
***Ehrlichia ruminantium*** **(16S rRNA)**		2 (14.29%)	1 (4.17%)			1 (1.30%)	MW467546	NR_074155, MH246936, U03776	100%
***Ehrlichia chaffeensis*** **(16S rRNA)**						2 (2.60%)		NR_074501	100%
***Anaplasma ovis*** **(16S rRNA)**		2 (14.29%)	2 (8.33%)	7 (15.56%)	1 (100%)	68 (88.31%)	MW467547-MW467552	MG869525	100%
***Candidatus*** **Anaplasma camelii** **(16S rRNA)**				1 (2.22%)			MW690202	MN630836	100%
***Rickettsia africae*** **(*ompB*)**		2 (14.29%)	4 (16.67%)				MW478135-MW478138	KU721071	100%
***Theileria ovis*** **(18S rRNA)**				1 (2.22%)		62 (80.52%)	MW467555-MW467561	MN712508, KX273858, MG738321	100%

## Data Availability

All nucleotide sequences generated in this study have been deposited in GenBank under the following accessions: MT896151-MT896154 (tick COI); MT895851-MT895865 (tick 12S rRNA); MT895169-MT89518116S (tick 16S rRNA); MT900489-MT900496, MW478135-MW478138 (*Rickettsia* spp.)*;* MT900497-MT900501 (*C. burnetii*); MT929189-MT929192 (*Ca.* E. regneryi); MT929193-MT929195, MW467546 (*E. ruminantium*); MT929188 (*E. chaffeensis*); MT929196-MT929197 (*Ehrlichia* sp.); MT929199-MT929201, MT929169-MT929177, MW690202 (*Ca.* A. camelii); MT929202 (*Anaplasma* sp.); MW541904-MW541911 (*Coxiella* endosymbionts); MW467555-MW467561 (*T. ovis*); and MW467547-MW467552 (*A. ovis*).

## Supplementary Materials

The following are available online at https://www.mdpi.com/article/10.3390/microorganisms9071414/s1, Table S1: Morphological and molecular identification of tick samples collected from camels in Marsabit, northern Kenya, February–March 2020, Table S2: Minimum infection rates for TBPs identified in ticks and camel blood samples according to sampling sites in Marsabit, northern Kenya, February–March 2020, Table S3: Numbers of sampled camels and sheep with the same TBPs in ticks and blood or in ticks or blood only, Marsabit, northern Kenya, February–March 2020.
