# Supplementary material for: Ticks and Tick-Borne Pathogens Associated with Dromedary Camels (Camelus dromedarius) in Northern Kenya
Source: Microorganisms. 2021 Jun 30;9(7):1414. doi: 10.3390/microorganisms9071414 (PMC8306667; doi:10.3390/microorganisms9071414)
Supplement: Supplementary file 1 [file microorganisms-09-01414-s001.zip › Table_S1.pdf]

Table S1: Morphological and molecular identification of tick samples collected from camels in Marsabit, northern Kenya, February-March 2020

Sample ID	Morphological ID	12S rRNA (% homology, GenBank Accession)	16S rRNA (% homology, GenBank Accession)	COI (% homology, GenBank Accession)	Consensus ID (GenBank Accession)[1]
**T1**	*Hyalomma dromedarii*	*Hy. dromedarii* (99.7%, MH094484)	*Hy. dromedarii* (100%, MN960589)	*Hy. dromedarii* (100%, MT107484)	*Hy. dromedarii* (12S: MT895851; 16S: MT895169; COI: MT896151)
**T108**	*Hyalomma dromedarii*	*Hy. dromedarii* (100%, MH094484 and KT391030)	*Hy. dromedarii* (100%, MN960589)	-	*Hy. dromedarii* (12S: MT895852; 16S: MT895170)
**T120**	*Hyalomma dromedarii*	*Hy. dromedarii* (100%, MH094484 and KT391030)	-	-	*Hy. dromedarii* (12S: MT895853)
**T208**	*Rhipicephalus pulchellus*	*Rh. pulchellus* (100% KY676841, AF150024)	*Rh. pulchellus* (100% MK774738)	-	*Rh. pulchellus* (12S: MT895854; 16S: MT895171)
**T209**	*Rhipicephalus pulchellus*	*Rh. pulchellus* (100% KY676841, AF150024)	*Rh. pulchellus* (100% MK774738)	-	*Rh. pulchellus* (12S: MT895855; 16S: MT895172)
**T275**	*Rhipicephalus camicasi*	*Rh. camicasi* (100% FJ536556, MH094506)	-	-	*Rh. camicasi* (12S: MT895856)
**T281**	*Rhipicephalus camicasi*	*Rh. camicasi* (100% FJ536556, MH094506)	-	-	*Rh. camicasi* (12S: MT895857)
**T303**	*Hyalomma impeltatum*	*Hy. impeltatum* (100% KX132904, MN315384)	*Hy. impeltatum* (100% MN394439)	*Hy. impeltatum* (99.2% KU130599)	*Hy. impeltatum* (12S: MT895858; 16S: MT895175; COI: MT896152)
**T318**	*Hyalomma impeltatum*	*Hy. impeltatum* (100% KX132904, MN315384)	*Hy. impeltatum* (100% MN394439)	*Hy. impeltatum* (99.2% KU130599)	*Hy. impeltatum* (12S: MT895859; 16S:

					MT895176; COI: MT896153)
**T362**	*Hyalomma rufipes*	*Hy. rufipes* (100% MN394460)	*Hy. rufipes* (100% MK737650, MK737649)	-	*Hy. rufipes* (12S: MT895860; 16S: MT895177)
**T403**	*Hyalomma rufipes*	*Hy. rufipes* (100% MN394460)	*Hy. rufipes* (100% MK737650, MK737649)	*Hy. rufipes* (99.7% JX049282)	*Hy. rufipes* (12S: MT895861; 16S: MT895178; COI: MT896154)
**T700**	*Amblyomma lepidum*	Am. lepidum (100% MK332385)	*Am. lepidum* (100% KP987777)	-	*Am. lepidum* (12S: MT895862; 16S: MT895179)
**T708**	*Amblyomma lepidum*	-	*Am. lepidum* (100% MK737651)	-	*Am. lepidum* (16S: MT895180)
**T710**	*Amblyomma gemma*	*Am. gemma* (99.7% KX377407)	-	-	*Am. gemma* (12S: MT895863)
**T749**	*Amblyomma gemma*	*Am. gemma* (99.7% KX377407)	-	-	*Am. gemma* (12S: MT895864)
**T852**	*Hyalomma truncatum*	*Hy. truncatum* (100% KU568497)	*Hy. truncatum* (99.4% KU130475)	-	*Hy. truncatum* (12S: MT895865; 16S: MT895181)

[1] accession numbers of samples deposited in GenBank as part of the present study
