# Supplementary material for: Ticks and Tick-Borne Pathogens Associated with Dromedary Camels (Camelus dromedarius) in Northern Kenya
Source: Microorganisms. 2021 Jun 30;9(7):1414. doi: 10.3390/microorganisms9071414 (PMC8306667; doi:10.3390/microorganisms9071414)
Supplement: Supplementary file 1 [file microorganisms-09-01414-s001.zip › Table_S2.pdf]

Table S2: Minimum infection rates for TBPs identified in ticks and camel blood samples according to sampling sites in Marsabit, northern Kenya, February-March 2020.

**Location**	**Host**	**No. of individuals**	**No. of pools**	***Coxiella burnetii***	***Coxiella* endo-symbionts**	***Candidatus* Anaplasma camelii**	***Anaplasma* sp.**	***Candidatus* Ehrlichia regneryi**	***Ehrlichia ruminantium***	***Ehrlichoia chaffeensis***	***Ehrlichia* sp.**	***Rickettsia africae***	***Rickettsia aeschlimannii***	***Paracoccus* sp.**
Laisamis	*Hy. dromedarii*	320	76	4 (1.4%)		9 (2.8%)		8 (2.5%)					1 (0.3%)	2 (0.6%)
	Hy. rufipes	364	96	6 (1.6%)		14 (3.8%)		13 (3.6%)					23 (6.3%)	8 (2.2%)
	Hy. impeltatum	39	14			1 (2.6%)		2 (5.1%)						
	Hy. truncatum	3	2											1 (33.3%)
	Rh. camicasi	23	8			2 (8.7%)								
	Camel blood	56		1 (1.8%)		52 (92.9%)		9 (16.1%)						
Kamboe	*Hy. dromedarii*	17	7			2 (11.8%)		1 (5.9)						
	Hy. rufipes	13	8			2 (15.4%)		2 (15.4%)						
	Am. gemma	19	12		1 (5.3%)	2 (10.5%)			2 (10.5%)			1 (5.3%)		
	Rh pulchellus	19	11	1 (5.3%)		3 (15.8%)					9 (47.4%)		1 (5.3%)	1 (5.3%)
	Hy. truncatum	2	1											
	Camel blood	16				16 (100%)		1 (6.3%)						
Shegel	*Hy. dromedarii*	179	34	5 (2.8%)		2 (1.1%)		2 (1.1%)						
	Hy. impeltatum	145	36			5 (3.4%)		3 (2.1%)					5 (3.4%)	2 (1.4%)
	Hy. rufipes	87	27	3 (3.4%)		4 (4.6%)		5 (5.7%)					9 (10.3%)	
	Hy. truncatum	13	4			1 (7.7%)								
	Rh. camicasi	38	10			3 (7.9%)								
	Am. gemma	3	3											
	Am. lepidum	3	3						1 (33.3%)					
	Camel blood	24		1 (4.2%)		20 (83.3%)		3 (12.5%)						
Korr	*Hy. dromedarii*	163	40	2 (1.2%)				3 (7.5%)					1 (0.6%)	
	Hy. impeltatum	37	13					1 (0.7%)					1 (2.7%)	
	Hy. rufipes	145	43	3 (2,1%)		1 (0.7%)		9 (20.9%)			1 (0.7%)		22 (15.2%)	
	Rh. pulchelus	2	1											
	Camel blood	56		7 (12.5%)		49 (87.5%)		13 (23.2%)						
Dabel	*Hy. dromedarii*	124	25			6 (4.8%)		3 (2.4%)						
	Hy. rufipes	39	15			2 (5.1%)		5 (12.8%)					8 (20.5%)	
	Am. gemma	8	8		1 (12.5%)	1 (12.5%)			4 (50%)			2 (25%)		1 (12.5%)
	Am. lepidum	41	20		3 (7.3%)	2 (4.9%)			4 (9.8%)			3 (7.3%)		
	Rh. pulchellus	28	17		2 (7.1%)	3 (10.7%)					5 (17.9%)			
	Camel blood	24				15 (62.5%)		4 (16.7%)						
Yaballo	*Hy. dromedarii*	37	13			3 (8.1%)		1 (2.7%)					1 (2.7%)	
	Hy. rufipes	22	10			2 (9.1%)		7 (31.8%)					3 (13.6%)	
	Am. gemma	26	16		1 (3.8%)	2 (7.7%)			4 (15.4%)			6 (23.1%)		

	Am. lepidum	29	15		1 (3.4%)	5 (17.2%)			3 (10.3%)		1 (3.4%)	3 (10.3%)		
	Rh. pulchellus	11	8			1 (9.1%)					3 (27.3%)			
	Rh. camicasi	3	3			1 (33.3%)							1 (33.3%)	
	Camel blood	24				14 (58.3%)								
Bori	*Hy. dromedarii*	15	10			1 (6.7%)		1 (6.7%)						
	Hy. rufipes	25	11					6 (24.0%)					6 (24.0%)	
	Am. gemma	29	16		5 (17.2%)	2 (6.9%)			2 (6.9%)			1 (3.4%)		
	Am. lepidum	7	5		1 (14.3%)	1 (14.3%)						1 (14.3%)		
	Rh. pulchellus	21	12								2 (9.5%)		2 (9.5%)	
	Camel blood	24				16 (66.7%)		3 (12.5%)						
Gola	*Hy. dromedarii*	18	10			1 (5.6%)		2 (11.2%)						
	Am. gemma	2	2		1 (50%)									
	Am. lepidum	65	28		3 (4.6%)	4 (6.2%)			6 (9.2%)			9 (13.8%)		
	Hy. rufipes	26	16			1 (3.8%)							5 (31.3%)	
	Rh. pulchellus	5	4		1 (20.0%)								1 (20.0%)	
	Camel blood	24				13 (54.2%)		3 (12.5%)						
Misa	*Hy. dromedarii*	13	4											
	Hy. rufipes	6	4			1 (16.7%)								
	Hy. truncatum	4	2										1 (25%)	
	Am. gemma	26	17		2 (7.7%)	1 (3.8%)			4 (15.4%)			2 (7.7%)		
	Am. lepidum	183	48		8 (4.4%)	8 (4.4%)			3 (1.6%)	2 (1.1%)		15 (8.2%)		
	Rh. pulchellus	4	4		2 (50%)									
	Camel blood	24				18 (75%)		2 (8.3%)						
Hula Hula	*Rh. camicasi*	8	3			2 (25%)								
	Am. gemma	4	4			3 (75%)						1 (25%)		
	Hy. dromedarii	1	1											
	Hy. rufipes	2	1											
	Rh. pulchellus	7	5	4 (57.1%)										2 (28.6%)
	Camel blood	8		1 (12.5%)		8 (100%)		3 (37.5%)						
Funanyatta	*Rh. pulchellus*	4	4		1 (25.0%)						1 (25.0%)			
	Hy. dromedarii	7	4			1 (14.3%)								
	Am. gemma	12	7		1 (8.3%)						1 (8.3%)	1 (8.3%)		
	Hy. rufipes	21	8										5 (23.8%)	
	Am. lepidum	2	1											
	Hy. truncatum	2	2											
	Camel blood	8				5 (62.5%)		1 (12.5%)						
Burgabo	*Hy. rufipes*	60	15			1 (1.7%)	1 (1.7%)	1 (1.7%)					6 (10.0%)	
	Hy. dromedarii	25	9											

	Hy. truncatum	1	1											
	Rh. pulchellus	3	1											
	Camel blood	8				7 (87.5%)								
