# Supplementary material for: Ticks and Tick-Borne Pathogens Associated with Dromedary Camels (Camelus dromedarius) in Northern Kenya
Source: Microorganisms. 2021 Jun 30;9(7):1414. doi: 10.3390/microorganisms9071414 (PMC8306667; doi:10.3390/microorganisms9071414)
Supplement: Supplementary file 1 [file microorganisms-09-01414-s001.zip › Table_S3.pdf]

Table S3: Numbers of sampled camels and sheep with the same TBPs in ticks and blood, or in ticks or blood only, Marsabit, northern Kenya, February-March 2020.

Camels (n =296)			
**Bacterial species**	**Same TBPs in ticks and in blood**	**TBPs in ticks only**	**TBPs in blood only**
Ehrlichia ruminantium	-	31 (10.5%)	-
Ehrlichia chaffeensis	-	2 (0.7%)	-
Candidatus Ehrlichia regneryi	10 (3.4%)	40 (13.5%)	37 (12.5%)
Ehrlichia spp.	-	16 (5.4%)	-
Candidatus Anaplasma camelii	63 (21.3%)	18 (6.1%)	170 (57.4%)
Anaplasma spp	-	1 (0.3%)	-
Rickettsia africae	-	32 (10.8%)	-
Rickettsia aeschlimannii	-	56 (18.9%)	-
Coxiella burnetii	1 (0.3%)	20 (6.8%)	9 (3.0%)
Coxiella endosymbionts	-	26 (8.8%)	-
**Sheep (n = 77)**			
Ehrlichia ruminantium	-	3 (3.9%)	1 (1.3%)
Ehrlichia chaffeensis	-	-	1 (1.3%)
Candidatus Anaplasma camelii	-	1 (1.3%)	-
Anaplasma ovis	5 (6.5%)	3 (3.9%)	63 (81.8%)
Rickettsia africae	-	3 (3.9%)	-
Theileria ovis	1 (1.3%)	-	61 (79.2%)
